# Protophones, the precursors to speech, dominate the human infant vocal landscape

**DOI:** 10.1098/rstb.2020.0255

**Published:** 2021-10-25

**Authors:** D. Kimbrough Oller, Gordon Ramsay, Edina Bene, Helen L. Long, Ulrike Griebel

**Affiliations:** ^1^ School of Communication Sciences and Disorders, University of Memphis, Memphis, TN, USA; ^2^ Institute for Intelligent Systems, University of Memphis, Memphis, TN, USA; ^3^ Konrad Lorenz Institute for Evolution and Cognition Research, Klosterneuburg, Austria; ^4^ Department of Pediatrics, Emory University School of Medicine, Emory University, Atlanta, GA, USA; ^5^ Marcus Autism Center, Children's Healthcare of Atlanta, Atlanta, GA, USA

**Keywords:** origin of language, vocal development, vocal learning, babbling, crying, laughter

## Abstract

Human infant vocalization is viewed as a critical foundation for vocal learning and language. All apes share distress sounds (shrieks and cries) and laughter. Another vocal type, speech-like sounds, common in human infants, is rare but not absent in other apes. These three vocal types form a basis for especially informative cross-species comparisons. To make such comparisons possible we need empirical research documenting the frequency of occurrence of all three. The present work provides a comprehensive portrayal of these three vocal types in the human infant from longitudinal research in various circumstances of recording. Recently, the predominant vocalizations of the human infant have been shown to be speech-like sounds, or ‘protophones’, including both canonical and non-canonical babbling. The research shows that protophones outnumber cries by a factor of at least five based on data from random-sampling of all-day recordings across the first year. The present work expands on the prior reports, showing the protophones vastly outnumber both cry and laughter in both all-day and laboratory recordings in various circumstances. The data provide new evidence of the predominance of protophones in the infant vocal landscape and illuminate their role in human vocal learning and the origin of language.

This article is part of the theme issue ‘Vocal learning in animals and humans’.

## 1. Background

The pursuit of roots for vocal learning in various taxa is hoped to provide perspective on how human vocalization evolved and eventually provided a basis for language. Our research has long sought to characterize the infrastructure for language through research on early vocal communicative development in humans, and we have argued for comparisons across species focused on similarities and differences at very early ages [1–3]. The earliest vocalizations in humans reveal foundations required for language to develop, foundations that are weak or missing in vocalizations of our non-human relatives. Because of the foundational nature of early development, we view comparisons in infancy as more instructive about the origin of language than comparisons of mature human language with vocal communication of other primates at any age.

A key to making quantitative comparison possible is to target similar, potentially homologous vocal types across species. We focus on three broad categories of sounds occurring in vocal communication of both human and other ape infants: (i) cries/screams, the salient distress sounds, (ii) laughs, the salient sounds of playfulness and positively valenced social connection, and (iii) other communicative or potentially communicative vocalizations, used in a variety of social and/or non-social circumstances, often at low intensity. This third category encompasses the speech-like sounds of human infancy, the ‘protophones’, including both non-canonical (e.g. squeals, growls and vowel-like sounds) and canonical babbling (consisting of canonical syllables such as ‘ba’ or ‘da’ and sequences, ‘baba’ or ‘dada’ and so on). In other ape infants, the third category includes utterances termed grunts, hoos, barks and so on.

Recent research has yielded surprises about the relative frequency of occurrence of these three types in humans. The expectation that cries are the foundation for vocalization and language in human infants [4] has been shattered—even from the first month, protophones outnumber cries by a factor of 5 to 1 or more [5,6]. Perhaps there exists a sort of attentional blindness whereby the saliency of infant cry draws us to note crying while failing to note the far more frequently occurring protophones. Even in infants born prematurely by more than two months, still in neonatal intensive care, protophones outnumber cries substantially [7].

The number of protophones produced by the human infant may seem astounding, having been determined by coding of randomly selected samples from all-day recordings to be approximately 3500 per day, a number that varies little across the first year [7]. The low frequency of occurrence of laughter will be addressed below to compare with the other types, using data not previously reported.

To our knowledge the only attempted direct comparison of frequencies of occurrence of the three broad types across species involved existing data on 37 human infants and data on three bonobo infants [8]. Human protophones were more than 10 times more frequent than any of the three vocal types in the bonobos. The predominance of the protophones has prompted speculations about their roots and their role in the origin of language. Protophones have been thought to be precursors to speech because they reveal the development of the acoustic features of speech sounds in systematic stages [9,10]. Also, protophones are functionally flexible, that is, each phonatory protophone type (e.g. squeals or vowel-like sounds) is used with different affective valences on different occasions, ranging from positive to neutral to negative, as judged by facial expression [6,11]. They can express delight, complaint, or simply interest in the sound itself on different occasions. This kind of flexibility is required in language, since all words or sentences can occur with and express any state of emotion or lack of it. It appears that functional flexibility is an absolute requirement of the sounds of spoken language, and thus we reason it may be necessary, if a species is to evolve toward language, to begin by evolving the ability and the inclination to produce vocalizations functionally flexibly.

The protophones appear to be produced largely endogenously. The rate of production is very high even when infants are alone. Perhaps even more surprisingly the great majority of protophones are not directed toward any listener even when caregivers are talking to babies [12]. An additional finding suggesting endogenous production is that congenitally deaf babies produce protophones at rates comparable to rates in hearing infants [13–16]. Furthermore, the protophones produced prior to the onset of canonical babbling (CB), appear to include the whole range of types (squeals, growls, raspberries, vocants and so on) heard in hearing infants [17]. The late onset of CB in deaf infants [18–20] does not necessarily suggest that CB is learned by imitation—we see no way to rule out the possibility that CB emerges as a self-organized product of prior protophone exploration.

Still, vocal interaction between caregivers and infants is clearly important in language development, and infants are motivated to attend to caregivers and to engage in systematic vocal exchanges [21–23] in addition to producing socially non-directed vocalization. Yet the predominantly endogenous driving of the protophones suggests that learning of vocal production categories during the first year may be primarily a result of self-organization, a consequence of infant exploration rather than of learning through input from caregivers.

If vocal development in the first year is indeed primarily self-organizing, then some traditional expectations need to be rethought. There has been considerable emphasis in language development research on acquisition by copying, with caregiver interaction and modelling driving imitation [24,25], a process whereby infants are presumed to absorb the native language's speech categories. Vocal imitation is thought to begin at birth [26], and the emphasis on parental ‘input’ and infant decoding and copying seems to supply, in this viewpoint, the primary method by which language units are learned [27]. Other research supports a theory of language acquisition based on perceptual learning from the environment of parental speech [28,29]. The literature on infant-directed speech (IDS) and its potential role in language acquisition is massive [30–36].

While vocal imitation is a logically necessary *capacity* for learning a lexicon, it is rare that infants actually produce immediate vocal imitation in the first year [37–39]. Attempts to experimentally show such imitation are fraught with ambiguities of interpretation as to whether the infant imitates or the parent induces and/or follows the infant's vocal explorations, a kind of following that can yield a false impression of representational imitation on the part of the infant [40]. A systematic attempt in our laboratory to identify cases of infant vocal imitation yielded no more than a handful of clear cases out of over 6000 utterances drawn from recordings of mother–infant interaction, with fewer than 5% showing *any* discernible imitation [41]. The cases that *did* show discernible imitation included apparent matching of subtle prosodic features subject to notable coder disagreement. Further, the mother's presumed model utterances might have constituted productions by her of sounds she knew to be in the infant's spontaneous repertoire, sounds that were likely to be produced by the infant with or without the model.

Imitation may be thought to be the source of novelty in infant sounds, but actual research does not support that novelty results from imitation. Experimental demonstrations of vocal imitation in humans [42,43] are essentially limited to illustrating that input can help direct infants in the first year toward the production of sounds already in their repertoires, not to novel sounds. We thus cannot rule out the possibility that sounds in infant repertoires (even canonical syllables) are indeed developed through self-organization rather than copying. Even purported ‘ambient language effects’ on babbling in the first year, e.g. [44], can be interpreted as representing modifications of usage of existing infant babbling syllables, rather than the acquisition of new ones. Thus while imitative *ability* is clearly required in language learning ultimately, it is unclear that imitative *acts* play much role, if any, in the first year.

Our line of reasoning supports a revision of the traditional view of vocal and language learning to envision infants as creators more than copiers. The present paper adds converging empirical data to the body of information reviewed above on the rate of occurrence of the three broad vocal categories (cries, laughs, protophones) of the human infant. The data are based on recordings that have been analysed from other perspectives in publications cited above and in papers currently in submission, but all analyses here are new. A particular novelty of these results is extensive longitudinal data on laughter rates, not previously reported, in spite of extensive interest in laughter as a basis for human vocal interaction [45–50]. Laughter is sharply different from cry in function, occurring almost exclusively in social interaction [49,51], a pattern that applies both in humans and in other apes.

The present study will:

1. for the first time provide longitudinal perspective across the first year for rate of occurrence of all three broad vocal types in human infants;
2. assess these rates based on both laboratory recordings and all-day home recordings; and
3. assess possible effects of interactive laboratory circumstances on the relative rates for all three vocal types.

The results will be evaluated in light of the role of protophones in language learning, as well as their implications for evolution. The results will also provide a more substantial frame of reference for more extensive planned quantitative comparisons across species in the near future.

## 2. Methods

### (a) The Atlanta data source

As part of a consortium effort to compare development in infants at risk and not at risk for autism, Emory University and the Marcus Autism Center in Atlanta, GA have for years been acquiring all-day recordings using the LENA battery-powered device [52,53] from infants across the first year. Mothers and infants were recruited through methods described extensively in a prior publication's electronic supplementary material [54]. Participation was always dependent on written informed consent from parents in accord with permission from the Emory University Institutional Review Board (IRB).

Here, we focus on 53 of those infants, for each of whom an average of 8.9 all-day recordings were obtained across the first year. All these infants have been confirmed to be typically developing, that is to have no developmental disabilities at 36 months. Human coding has produced data on rates of production of the three broad categories of infant sounds for each recording. Human coding was conducted in Memphis in a collaboration between the institutions with IRB permission from both Emory University and the University of Memphis.

### (b) The Memphis data source

In a separate effort, longitudinal research on 12 human infants has been conducted in Memphis over the past 10 years. Again, recruitment was conducted for pregnant women with approval from the University of Memphis IRB, and written informed consent was provided by the parents. Typical development was confirmed using developmental milestone questionnaires. The Memphis research has produced two kinds of data relevant to the present report: first, each infant was recorded in a laboratory setting across the first year, and second, each infant was recorded using the same LENA all-day recording method as in Atlanta. For each of the 12 infants, both laboratory and all-day home recordings yielded data at six ages. Again, human coding in Memphis provided data on the three broad groupings of infant sounds.

### (c) All-day recordings

The battery-powered LENA recorder can be placed in the vest pocket of infant clothing to produce up to 16 h of continuous audio at 16 kHz. The microphone is nominally 5–10 cm from the infant's mouth, offering high signal-to-noise ratio for the infant voice under most circumstances.

The device has been used in many thousands of recordings since 2007–2008, when it first became available [55]. It has generated a new perspective on vocal development and caregiver–infant interaction by opening the door to more representative sampling than has been available in prior research. Based on data in submission for publication, the new perspectives include, for example, apparently lower rates of CB (the most advanced protophone type) in the all-day LENA recordings than have been reported in short-term laboratory recordings, as well as notable differences between the patterns of caregiver–infant vocalization observed in standard laboratory recordings and LENA recordings [8]. Importantly, parents have been shown to produce several times more IDS in the standard recording situations than they do in the presence of wakeful infants in randomly sampled segments from all-day recordings in the home. Results below will provide comparisons suggesting differences in protophone rates as well across all-day recordings and laboratory recordings.

The LENA Foundation's automated analysis system has been widely used in research on vocal development [56–58], but the work reported here is based on the more labour-intensive method of human coding of randomly sampled 5 min segments across each recording. Human coding is the gold standard for the development of automated analysis of vocalizations, and the rate of infant laughter is not counted directly by the LENA automated system.

In both Atlanta and Memphis, parents placed a fully charged and activated recorder in a vest worn by the infant at wake-up time and left it running until bed time. During naps or bath time, the recorder was removed from the vest and left running in a location as near the infant as practical and was then placed back in the vest. The instructions encouraged parents to record in the home with no changes in the normal pattern of interaction and caregiving. The precise procedures for recording are described in detail in prior publications [7,54].

### (d) Laboratory recording method

The 12 infants in the Memphis study were also recorded across the first year in a laboratory designed to resemble a child's playroom. There were eight cameras, one placed high and one placed low in each corner of the room. High-fidelity wireless microphones were worn in an infant vest and on the parent's lapel, recording at 48 kHz, with video subsequently synchronized with frame-level accuracy to the high-fidelity audio from the two microphone channels. Two channels of video (from the eight cameras) were selected at each point in time by staff in the adjacent control room, providing one view of the infant and another of the interaction.

The laboratory recordings were typically 1 h in duration although sometimes the sessions were broken up into smaller segments with temporary interruptions to accommodate feeding or infant discomfort. Scheduling was designed to avoid times when an infant would be likely to fall asleep, but on occasion, especially at the youngest ages, sleep also interrupted recordings, which had to be either rescheduled or started again after the infant woke up. The protocol for recording involved three segments of nominally 20 min each. These were roughly counterbalanced in order of occurrence.

(1) In the No Adult Talk circumstance, the parent was in the room, reading or engaging in another silent activity while the infant was nearby, often playing.
(2) In the Adult to Adult Talk circumstance, the infant was nearby in the room, while the parent engaged in a verbal interview with a staff member of the project.
(3) In the Parent Infant Talk circumstance, parent and infant interacted playfully, with considerable IDS.

Data on rates of the three broad vocal types have not been previously reported for these three circumstances. The laboratory recordings at the same six ages as for the LENA recordings were human coded in Memphis according to the procedures described below.

### (e) Sample selection for coding

The 21 and 24 randomly selected 5 min segments were extracted from each all-day recording from Atlanta and Memphis, respectively. These segments were subject to human coding as specified below. After coding, some segments were excluded from analyses because the infant was deemed to be asleep by the coders, yielding 7387 five-minute segments from the 474 all-day recordings of the 53 Atlanta infants and 1185 from the 69 all-day recordings of the 12 Memphis infants. The 67 human-coded Memphis laboratory recordings were approximately 1 h each: all 12 infants had recordings at five of the six ages across the first year, but only seven had recordings at the youngest age. The laboratory recordings yielded 59, 66 and 64 sessions of data for the No Adult Talk, Adult to Adult Talk and Parent Infant Talk circumstances, respectively.

### (f) Coding

Coding determined counts for protophones, cries, whimpers and laughs, which together accounted for 99% of all utterances. The three phonatory protophone types that were coded for inclusion in the analysis (squeals, growls and vowel-like sounds, including utterances with and without canonical syllables) were collapsed together. Cries and whimpers (for the definition of the distinction, see [59]) were also collapsed into a single distress category.

Protophones (both canonical and non-canonical) are largely produced spontaneously: no particular emotional state or stimulus is needed to produce them. Thus, they provide a basis upon which speech development depends since it must be possible to produce any element of speech in any emotional state as well as in a state of affective neutrality or pure interest in self-produced sound. Coders were encouraged to work intuitively in differentiating protophones from cry/whimper and laughter. Training criteria and coding instructions have been provided in detail in prior studies [7,54]. Coder agreement on the distinctions is presented below.

Protophones, cry/whimper and laughter were all counted in accord with a ‘breath group’ criterion [60]: each voiced period produced on a single egress was counted as one utterance. Thus, all three utterance types were counted in a similar way, breaking cry/whimper and laughter into utterances of roughly similar dimensions to protophones.

After coding each 5 min segment, coders responded to the following questions (among others not relevant here): (1) Did any other person talk to the baby? This could be the parent or another adult or child. (2) Do you think the baby was alone in the room? and (3) Do you think the baby was asleep? The questions were answered on a 5-point scale, where 1 indicated Never, 2 Some of the time, 3 About half the time, 4 Most of the time, and 5 The entire time.

Coders for both all-day and laboratory recordings were 16 normally hearing female students from the University of Memphis School of Communication Sciences and Disorders, who had been trained in phonetic transcription during their programme of study. The additional six- to eight-week training for the coding of infant vocalizations is described in detail in prior publications [7,54]. The set of segments corresponding to recordings from each infant was assigned to a single coder. The protocol specified that coders should work through the entire dataset for each infant to which they had been assigned before proceeding with the next infant. Coding of each recording was completed before coding of another recording was begun, and the (21 for Atlanta or 24 for Memphis) 5 min segments were coded, and questionnaires were answered for each segment in the chronological order in which they had occurred during the recording day.

### (g) Coder agreement

Each of 523 five-minute segments was coded independently by two of the coders—12 coders participated in this agreement study. Each coder was semi-randomly assigned to segments from six different ages and at least four different infants for the agreement coding. The correlations between counts for the coders were: protophones: *r* = 0.84, *ρ* = 0.91; cry/whimpers: *r* = 0.94, *ρ* = 0.77; laughs: *r* = 0.89, *ρ* = 0.67. Restricting the data to the second half-year only, when laughter is much more common than earlier, the 293 five-minute segments showed correlations of: protophones: *r* = 0.84, *ρ* = 0.92; cry/whimpers: *r* = 0.84, *ρ* = 0.77; laughs: *r* = 0.93, *ρ* = 0.73. There are much additional data on agreement among coders in prior publications [7,54].

## 3. Results

**Figure 1.**
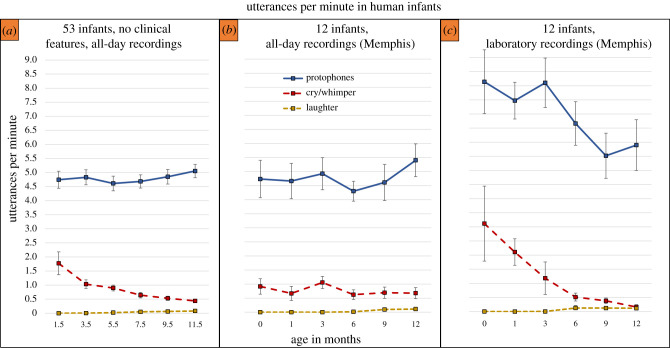
Rates of protophones, cry/whimpers and laughs in human infants across the first year. (*a*) Based on all-day recordings of 53 infants determined to be typically developing in the Atlanta sample, we found protophones were massively more frequent than cry/whimpers, and cry/whimpers were massively more frequent than laughs. Standard error bars illustrate the reliability of these differences, although the standard errors were so small for the laughs that they are contained within the square markers. (*b*) Based on all-day recordings of 12 typically developing infants in the Memphis sample, the patterns were similar to those from the Atlanta sample, although given the smaller sample size, the error bars are larger. (*c*) Based on laboratory samples for the same 12 infants in Memphis, the patterns confirm those from the all-day recordings, although rates of protophones and cries were lower at the older ages than the younger ones in the laboratory. Means and standard errors for figure 1 were computed at the infant level.

Figure 1*a* shows relative rates of protophones, cry/whimpers and laughs in the Atlanta data, with laughter showing rates so low that the divergence from zero is hard to discern on the graph prior to the middle of the first year, not surprisingly since laughter in human infants has an onset at three to four months [49]. In the second half-year, >1400 laughs occurred during wakeful segments, but cry/whimpers were >8 times more frequent (>12 000) and protophones approximately 74 times more frequent (>106 000) than laughs. Thus, laughs occurred on average about 3.8 times per hour in the second half-year, cries 34 times per hour and protophones nearly 292 times per hour.

The Memphis data based on all-day recordings are displayed in figure 1*b*, supporting the basic patterns of the larger Atlanta sample. In the second half-year laughs were infrequent (*N* = 228) compared with protophones (14 658 or 64 times more frequent than laughs) and cry/whimpers (2043 or 9 times more frequent than laughs).

Recording type played a role in the frequency of occurrence, as illustrated in figure 1*c*; the 12 Memphis infants in the laboratory setting produced more protophones than in the all-day recordings. They also produced more protophones and more cry/whimpers early in the year than later. Yet even in the laboratory setting, laughs were very infrequent compared with the other vocal types. In the second half-year, protophones (13 396) were 50 times more frequent than laughs (*N* = 268), and cry/whimpers (784) were 2.9 times more frequent than laughs. The reduction in the frequency of occurrence across age for protophones in the laboratory recordings (figure 1*c*) may be due to the greater mobility of infants, who in the second half-year tended to crawl or walk about the playroom finding toys and other objects to explore.

**Figure 2.**
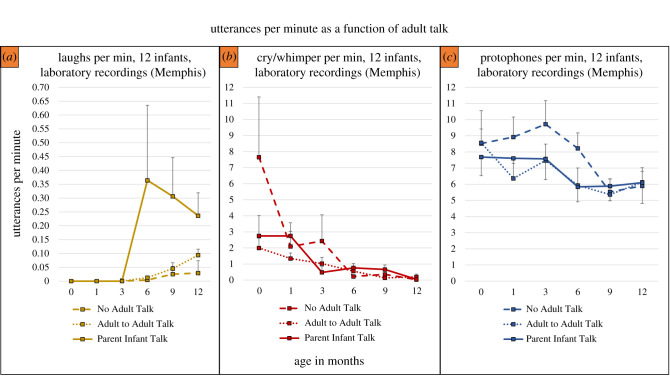
Rates of the three vocal types based on three different laboratory recording protocols. (*a*) Laughs occurred infrequently in all three protocols in the first three months. Although the frequency was very low compared with cry/whimpers and protophones in the second half-year (note the *y*-axis scale differences for (*a*) versus (*b*) and (*c*) to allow better visualization of differences in laugh rates across age), the rates of laughter were, as expected, at their highest in the Parent Infant Talk circumstance (infant-directed speech). (*b*) Cry/whimpers were far more frequent than laughs at age 0 and up to and including three months (because laugh onset tends not to occur until about that age), but rates were more comparable in the second half-year. Cry/whimper rates based on the laboratory counts should, however, be interpreted in light of the fact that the recordings were sometimes interrupted to soothe a crying or fussing infant. The high rate of cry/whimper during the No Adult Talk circumstance at zero months appears to have been the result of infant distress at being left nearby but unattended, which resulted in either the parent deciding to hold the infant during No Adult Talk or interruption of the recording to calm the infant. (*c*) Protophone rates were higher in the laboratory than in the all-day recordings though they tended to fall across the first year. Note that Parent Infant Talk did not correspond to notably higher rates of protophones than in the other circumstances, a fact we interpret as corresponding to the largely endogenous nature of protophone production. Means and standard errors for figure 2 were computed at the infant level.

The data in figure 2 present a breakdown of figure 1*c* in terms of the three laboratory recording circumstances. Figure 2*a* illustrates a salient effect of parent–infant interaction, where laughter, as expected, was most frequent in the second half-year during the Parent Infant Talk sessions. The existence of the small amount of laughter in the No Adult Talk and Adult to Adult Talk sessions may be due to the fact that parents occasionally violated recording protocol and attended to infants briefly. Note that the scales are different for figure 2*a* versus 2*b* and 2*c* to make it possible better to visualize the differences in the low laugh rates across ages. Regardless of circumstances or age, protophones (figure 2*c*) were >14 times more frequent than laughs in the laboratory at every age and every circumstance. The very high rate of cry/whimper at the youngest age (figure 2*b*) in the No Adult Talk circumstance can be attributed, we think, to infant protest at being left in a crib or stroller with little or no adult attention—mothers did not allow the crying to go on too long, choosing to hold the infant while reading if the infant persisted in crying. Figure 2*a* shows that at the latest ages, laughter proved to be about as frequent as cry/whimper in the Parent Infant Talk circumstance.

We also considered rates of laughter occurring in the all-day recordings as a function of the amount of IDS and whether the infant was alone during the 5 min segments as indicated by the questionnaire items. Results for the second half-year support the long-recognized fact that laughter is a social phenomenon, with 6.6 times more laughter occurring with IDS than without IDS, and 8.8 times more laughter when infants were not alone than otherwise. At the same time, also in the second half-year, even in segments when someone was talking to an infant, the rate of laughter (0.098 per minute) was very small compared with protophone rates (4.8 per minute).

## 4. Discussion

### (a) The high frequency of protophones and the endogenous nature of vocal development

The massive rate of protophone production as seen robustly in a variety of circumstances in the present results as well as prior studies cited above, along with the fact that protophones are largely directed to nobody from the beginning of human life and throughout the first year, compel us to recognize that the activity is predominantly generated endogenously. Laughter and cry/whimper, on the other hand, are generated primarily in situations of either social play or distress, but at rates that are much lower than for protophones (figures 1 and 2). These more emotionally grounded signals play the same kind of role in humans that similar vocalizations play in other mammals and prominently in the great apes. But protophones are at best minimally present in other apes [8], and to the extent that they may occur, they have never been shown to exhibit the exploratory characteristic that has been observed as the predominant mode of production of human protophones.

We have long argued that in the absence of the ability to produce protophones, the development of language would be impossible [1,3]. The reason is simple and logical: language elements can be produced in any circumstance of emotion or illocutionary intent—the word ‘apple’ can be produced to complain, to request, to name, to correct, to criticize, to teach, or to practise the pronunciation of the word, and in any state of pleasure or displeasure. If it were not so, ‘apple’ would not be a word and could not pertain to the lexicon of any language. Thus, the ability to produce a set of particular sounds freely in any emotional state is clearly a foundation without which learning to use a word would be impossible. We call this capability to produce particular sounds in any emotional state ‘vocal functional flexibility’ (VFF), and have proven it to be present extensively in human infant protophones in the first months of life [6,11]. Laughs and cry/whimpers in infancy do not show VFF.

### (b) The infrequency of laughter

The salience of laughter events that sometimes occur repeatedly in playful interactions between parents and infants (in peekaboo, for example) provides intuitively persuasive evidence that bonding and social learning may be richly served by such joyful interactions. Yet the infrequency of laughter occurrences based on this extensive sampling from all-day recordings, a rate at least 50 times lower than that of protophones, is surprising. The rates of laughter reported here for naturalistic laboratory recordings are *not* low compared with rates that have been reported previously based on experimental observations of parent–infant interaction. In fact, the rates in the second half-year for the Parent Infant Talk circumstance in the Memphis data appear to be a little higher than those reported in the most comprehensive previous study we know of reporting infant laughter rates [48]. In the presence of a mother not engaged in IDS, the cited study reported laughter rates lower than in those of the present data.

Infant laughter is salient not only in humans but also in other apes [61]. In the only direct quantitative comparison we know of across human and non-human ape infants [8], we found that three bonobo infants laughed during rough and tumble play or tickling and that laughter appeared to be the most frequent type of vocalization in the bonobo infants. The sample size was insufficient to make useful statistical comparisons of rates of laughter in the human and bonobo infants, but protophones in the humans were far more frequent than laughter or cry/scream in either case. Playful laughter has been reported for all the great apes and for many mammals [61,62], and has been speculated to provide a phylogenetic platform for the evolution of language [46,47,63]. Yet its occurrence in human infants was shown here to be remarkably rare, especially when compared with the protophones.

### (c) Variations in rates of the three broad categories of vocalizations across circumstances of recording

The results of the present work provide the strong suggestion that, while protophones are robustly the most frequent vocal type in all circumstances of recording that have been studied thus far, there are notable variations in rates of production for all three vocal types depending on circumstance. Laughter predictably occurs almost exclusively in social interaction (figure 2*a*). Both protophones and cry also appear to vary by circumstance, with more protophones and cries in the laboratory circumstance than the all-day recordings during the first half-year (cf. figure 1*b*,*c*). A number of factors that are not easily controllable in such observational research could play roles in these apparent variations, including but not limited to: (i) parents may exert more effort eliciting vocalization during laboratory recordings; (ii) crying rates may be high in the early months of laboratory recordings because the setting is unfamiliar to the infants; (iii) high crying rates at the youngest age may be due to parents' having been instructed to try not to respond to their infants during the No Adult Talk condition (cf. figure 2*b*); and (iv) infants may be more awake and alert on the whole during laboratory recordings than during all-day recordings (cf. figure 1*b*,*c*). The data suggest we cannot rule out the possibility that babies produce most protophones during the Parent Infant Talk circumstance (cf. figure 2*c*). This result confirms a similar outcome with a separate group of infants evaluated in laboratory recordings by Iyer *et al*. [64]. The converging evidence appears to further support the conclusion that protophone production is fundamentally endogenous rather than being driven by vocal interaction.

### (d) Fitness signalling and the origin of language

Why, then, do protophones exist at all? And why do they occur so frequently compared with crying and laughter? The questions are not trivial because it can be assumed that the ability to produce sounds with VFF must have preceded the origin of vocal language. Consequently, at their earliest appearance in hominin evolution, vocalizations with VFF must have been selected for in accord with pressures that had nothing to do with language, which did not yet exist.

The evolutionary origins of laughs and cry/whimpers, in contrast, fit the more standard mould of presumable selection pressures. Both these types of vocalizations express definable emotional states and serve definable and relatively consistent functions that have direct potential benefits at the moment they are produced. Cry/whimpers signal need for care, and laughter signals playful social connection. It seems straightforward to postulate that mammals, being dependent on maternal care, are under selection pressure to have the ability to produce these kinds of sounds as needed. Interestingly, there has been considerable speculation about human infant cry as a fitness signal [65], but only more limited and recent speculation about protophones as fitness signals.

Protophones are different from cry and laughter because they do not have a fixed valence nor a predominant immediate social function that could have been the basis for selection. Even a comfortable infant who is entirely alone produces massive numbers of protophones, and even if parents are talking to an infant, most of the infant's protophones are not directed to anyone [12]. So, we reason, the predominant function of the protophones must be based on advantages that do not usually accrue in the immediate context of their production. Rather, we argue, the protophones predominantly supply information about infant wellness even to caregivers who are busy doing something else nearby.

This kind of fitness signalling has been argued to be particularly important to human infants for two reasons. First, human infants and their hominin predecessors are and were more altricial than other apes, with much longer developmental periods of helplessness and need for provisioning by others [66]. Thus, pressure on signalling their wellness may have resulted in the ancient hominin infant vocal system being selected for high activity, driven by the same motivational/emotional system that generates exploration with the hands in other baby primates. We presume that sounds produced by the infant hominin's own phonatory system came thus to be objects of exploration and play [67,68]. The capability and inclination to produce these sounds, and thus to indicate wellness, presumably put them at an advantage with respect to other hominin infants in the competition for investment by provisioning from caregivers and in the competition to be kept rather than abandoned in times of stress.

A second reason that the pressure on vocal fitness signalling may have been particularly high in hominin infants is that ancient hominin groups were larger than those of other apes and increasingly so over the evolution of the hominins [69]. These larger groups were also increasingly cooperative breeders, with infants being cared for and provisioned not just by mothers, but by alloparents, a pattern of rearing seen strongly in just one other group of primates, the New World callitrichids. Notably, this is the only other group of primates known to engage in ‘babbling’ in infancy [70,71]. We reason, along with others, that the pressure on vocal fitness signalling runs deep in the hominin line both because of altriciality and because of cooperative breeding, given that infants could profit from broadcasting fitness indicators in the competition for care from a variety of alloparents [72].

There are many fitness indicators: colour of the skin, vigour of movement, ability to raise the head, ability to move the fingers, and so on. We concur with authors who have argued that human communication is multimodal (involving facial expression and gesture as well as vocalization), and we presume that the protophones may thus have emerged in the context of pressures on several modes of expression that may also have involved fitness signalling. Current research in our laboratory is addressing relative rates of facial expression, gesture and vocalization in the first year. All these factors can play roles in how caregivers of various mammalian species determine their investments in their young. The protophones offer a special leg up on fitness signalling, however, because they can occur even when the potential caregivers are not attending to them, for example, after putting the infant down during foraging. We reason that the value of vocal signals may be recognized, even if semiconsciously, accumulating in the awareness of the caregiver, who may provide benefit to the infant much later.

What of other possible selection pressures that might favour endogenous production of protophones? One possibility is that although language did not exist when the first protophone-like utterances began to appear, perhaps there was pressure for each individual infant to prepare through vocal practice for affective and fitness signalling vocal communication with potential allies and mating partners later in life. The problem with such a suggestion in our opinion is that it runs foul of the principle that natural selection does not see into the future, that evolved capacities (and by implication developed capacities) must serve some selectable function at the time they first appear [73]. Of course, there could ultimately exist a role for infant practice in vocalization for later vocal capabilities, but that role would have to have evolved secondarily, as an advantage built upon the primary advantage of exploratory vocalization.

Our proposal does not suggest that protophones constitute language. Rather, we propose that the ability and inclination to produce protophones supply a platform on which later development can build. Further, ancient hominin infants, according to our proposal, were selected to produce protophone-like sounds first, and later came under additional natural selection pressures for more elaborate communication. Vocal language would not be possible without a foundation of functionally flexible vocalization, but much remains to be evolved and developed beyond the achievement manifest in the protophones.

## Data accessibility

Data are provided as the electronic supplementary material [74].
